# Breakthrough applications of porous organic materials for membrane-based CO_2_ separation: a review

**DOI:** 10.3389/fchem.2024.1381898

**Published:** 2024-03-21

**Authors:** Yan Cao, Ali Taghvaie Nakhjiri, Mahdi Ghadiri

**Affiliations:** ^1^ School of Computer Science and Engineering, Xi’an Technological University, Xi’an, China; ^2^ Department of Petroleum and Chemical Engineering, Science and Research Branch, Islamic Azad University, Tehran, Iran; ^3^ Institute of Research and Development, Duy Tan University, Da Nang, Vietnam; ^4^ The Faculty of Environment and Chemical Engineering, Duy Tan University, Da Nang, Vietnam

**Keywords:** porous organic materials (POMs), functionalization, CO_2_ separation, membrane, chemical characterization

## Abstract

Over the last decades, porous organic materials (POMs) have been extensively employed in various industrial approaches including gas separation, catalysis and energy production due to possessing indisputable advantages like great surface area, high permeability, controllable pore size, appropriate functionalization and excellent processability compared to traditional substances like zeolites, Alumina and polymers. This review presents the recent breakthroughs in the multifunctional POMs for potential use in the membrane-based CO_2_ separation. Some examples of highly-selective membranes using multifunctional POMs are described. Moreover, various classifications of POMs following with their advantages and disadvantages in CO_2_ separation processes are explained. Apart from reviewing the state-of-the-art POMs in CO_2_ separation, the challenges/limitations of POMs with tailored structures for reasonable application are discussed.

## 1 Introduction

Over the last decades, significant increment in the anthropogenic industrial-based release of carbon dioxide (CO_2_) greenhouse gas has exacerbated the risk of serious air pollution and unfavorable climate changes like global warming, unbalanced pattern of precipitation and sea-level rise, which not only endanger the humans’ health but also negatively affect economic systems (Nakhjiri et al., 2018/11; Carnicer et al., 2022; Huhe et al., 2023). The maximum permittable value of CO_2_ in natural gas for commercial natural gas is 2.5% (Mazzetti et al., 2014). Higher value of this water-soluble chemical compound in natural gas can result is different detrimental effects like corrosion of pipe lines and reduction of gas heating value (Nakhjiri et al., 2020). Therefore, development of cutting-edge, environmentally-friendly, cost-effective and breakthrough technologies to increase the separation rate of CO_2_ from gaseous mixtures is of prime importance in industry. Table 1 aims to present detailed information about CO_2_.

**TABLE 1:** Detailed information about CO_2_ (Stein, 2022; Guais et al., 2011; Vesovic et al., 1990; Cao et al., 2021/09).

Molecular structure	Molar mass (mol)	Appearance	Solubility in water	Toxicity
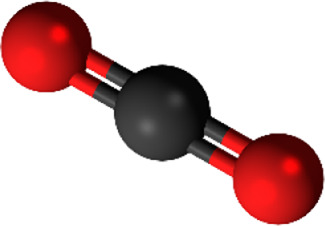	44.09 g^−1^	Colorless gas	1.45 g/L (at 25°C)	Headaches
				Dizziness
				Difficulty in breathing
				Sweating
				Increased heart rate
				Asphyxia
				Convulsions

In recent years, membrane-based separation technology has been of prime attention in various situations such as CO_2_ removal, air dehumidification, solvent extraction and precious metals recovery due to its noteworthy advantages such as energy efficiency and environmental benefits (Cannone et al., 2021; Cao et al., 2021; Cao et al., 2021/04; Taghvaie Nakhjiri et al., 2022; Ghadiri et al., 2020; Pishnamazi et al., 2020/09). Appropriate microporous membranes should possess great permeability and selectivity toward separation of specific gases. Despite the significant modification of membranes using physical techniques (i.e., fabrication route and membrane configuration), the advancement of more efficient materials with brilliant separation capabilities has been of great attention among academic researchers (Zou and Zhu, 2018; Babanezhad et al., 2020). Polymers (i.e., cellulose acetate (CA), polyamides (PAs), and polyimides (PIs)) are known as the most prevalent employed membrane materials in industrial gas separation applications, which have been prosperously commercialized since the 1980s (Zhang et al., 2013; Baker and Low, 2014). Generally, polymeric membranes with high selectivity have low permeability, and *vice versa*. Dense or low-porous phase of polymeric membranes is the reason of justifying this trade-off relationship, which is able to be empirically and theoretically confirmed by Robeson and Freeman, respectively (Freeman, 1999; Robeson, 2008). Porous organic materials (POMs) are developing as an emerging solution for the issue. POMs are known as the hydrocarbons including pores/voids in the microporous region. Their structures are created by organic moieties adjoined via vigorous covalent bonds, often eventuating in ordered and rigid structures. Porous Aromatic Frameworks (PAFs), Conjugated Microporous Polymers (CMPs), Hyper-Cross-Linked Polymers (HCPs), Polymers of Intrinsic Microporosity (PIMs), Covalent Organic Frameworks (COFs) and Covalent Triazine Frameworks (CTFs) are regarded as the certain classifications of the POMs (Nakhjiri and Roudsari, 2016; Das et al., 2020; Krishnaraj et al., 2020; Lee and Cooper, 2020; Lee et al., 2020; Pishnamazi et al., 2020; Ramezanipour Penchah et al., 2020). Based on the suggestion of International Union of Pure and Applied Chemistry (IUPAC), those pores with persistent connection routes with the external surfaces of the porous structure are called open pores, while those pores that are segregated from others are considered as the closed pores (Rouquerol et al., 1994). The open pores possess brilliant capabilities to be applied in fluid dynamics and gas separation, and therefore, have been of great interest among numerous chemists and chemical engineers all over the world (Rouquerol et al., 1994). The classification of the POMs based on their pore size (according to the IUPAC recommendation) is as follows (Das et al., 2017):

A) Microporous POMs with pore size lower than 2 nm;
B) Mesoporous POMs with pore size 2–50 nm;
C) Macroporous POMs with pore size higher than 50 nm.

**FIGURE 1:**
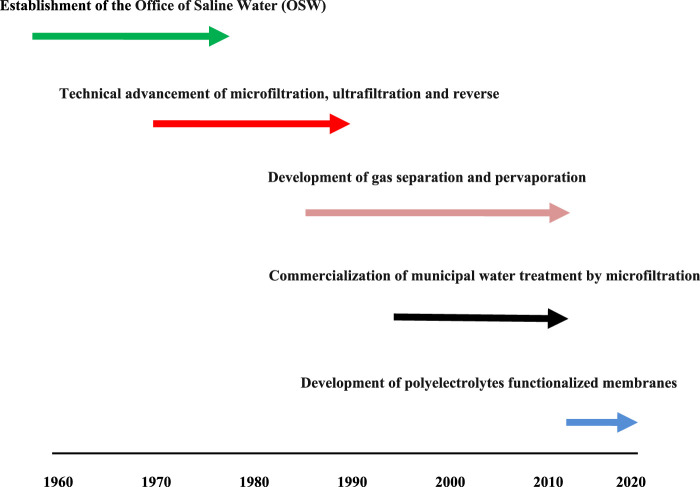
The development of the membrane separation industry, 1960–2020. Adopted from (Baker, 2004; Li et al., 2020).

Figure 1 presents the development of the membrane separation industry.

Most of POMs have noteworthy characteristics such as great surface areas, appropriate thermal stability and negligible framework density. The aforementioned features have made them promising for application in gas separation, catalysis, and biomedical systems (Ding and Wang, 2013; Zou et al., 2013; Little and Cooper, 2020). Therefore, significant endeavors have been made to synthesize and consequently characterize the POMs with disparate chemical structures. POMs-based membranes may act as a novel classification of molecular sieves thanks to their high porosity and small pores at molecular levels. Therefore, great separation efficiency of gas molecules can be expected if POMs are correctly processed for the membranes (Budd and McKeown, 2010; Marjani et al., 2021).

The main objective of this review study is to discuss the current advancements in the application of POMs in CO_2_ separation. Various classes of POMs such as HCPs, PIMs, COFs, CMPs, CTFs and PAFs following with their advantages and disadvantages in CO_2_ separation processes are subjected. In addition to reviewing the breakthrough applications of MOFs in CO_2_ separation, the challenges/restrictions towards the true understanding of POMs with tailored structures for reasonable applications are discussed.

## 2 Classification of POMs

Here, different types of POMs accompanying with the characteristics of each classification are described.

### 2.1 HCPs

HCPs are known as one of the most important amorphous polymers possessing great surface areas and low densities, which are synthesized applying disparate chemical procedures (i.e., Friedel − Crafts alkylation chemistry) from other POMs. Moderate reaction conditions, cheap reagents, and ease of scale up are the privileges of HCPs, which make them promising in gas separation, catalysis and removal of aromatic molecules from water (Wood et al., 2008; Xu S. et al., 2013; Masoumi et al., 2020). Wang et al. evaluated the adsorption efficacy of CO_2_ applying different types of HCPs functionalized by ethylenediamine (EDA). Based on the influence of amine functionalization, they perceived that the selectivity of amine functionalized HCPs (HCPs-A) significantly enhanced and reached to 85.71 and 8.12 for CO_2_/N_2_ and CO_2_/CH_4_ gaseous mixtures due to increased specific surface area and microporosity (Wang et al., 2020).

The prevalent techniques applied for the synthesis of HCPs are (Xu S. et al., 2013; Tan and Tan, 2017; Yang et al., 2020):

A) Post-cross-linking (PCL) of polymers;
B) Direct one-step polycondensation (DOP);
C) Application of external crosslinkers (ECLs).

**FIGURE 2:**
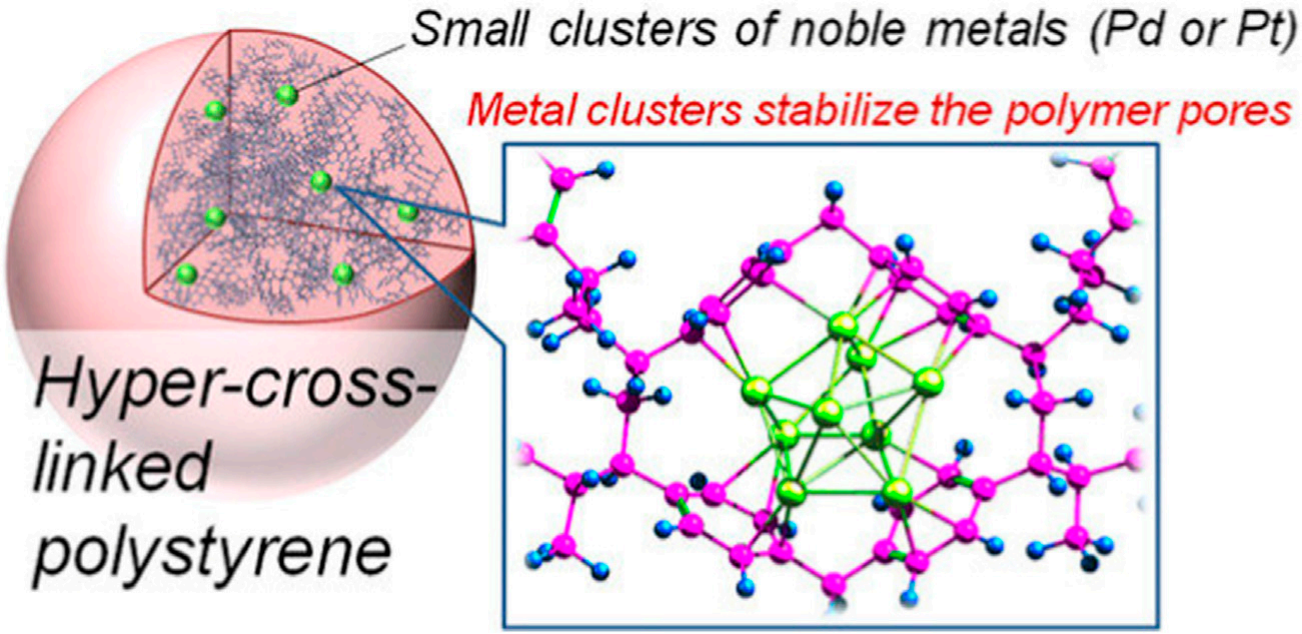
Illustration of hyper cross-linked polystyrene (Bykov et al., 2021).

The PCL procedure starts by polymeric precursors’ dissolution in solvent. Whenever the swelling process initiates, the polymeric chains are released from a tangled or twisted state and the free area between them is occupied by the solvent. Then, the polymeric chains are exposed to cross-linking process. After solvent removal, the polymeric chains are disassociated using the cross-links, finally eventuating in the preparation of an inter-linked porous polymer. Discovery of Davankov resins has eventuated in an instant advancement in the design, characterization, synthesis and application of HCPs. The most noteworthy privilege of DOP is the direct application of commercial-based accessible polymeric products as precursors for PCL process (Tan and Tan, 2017). Various synthetic procedures consisting of self or co-condensation of chloromethyl/hydroxymethyl-based monomers have been offered for the appropriate synthesis of HCPs. However, all of the abovementioned procedures suffer from certain drawbacks like the need of great amounts of organic reagents and solvents following with energy cost for further purification steps. An outstanding development is ECL strategy in which active formaldehyde dimethyl acetal is used as an external crosslinker to blend simple aromatic components such as benzene with rigid methylene bridges by means of the anhydrous FeCl_3_ catalyzed Friedel–Crafts reaction (Tan and Tan, 2017). Application of external cross-linkers as the third synthesis procedure, which has resulted in increasing the variety of HCPs (Germain et al., 2007). Schematic demonstration of hyper cross-linked polystyrene is presented in Figure 2.

### 2.2 PIMs

**FIGURE 3:**
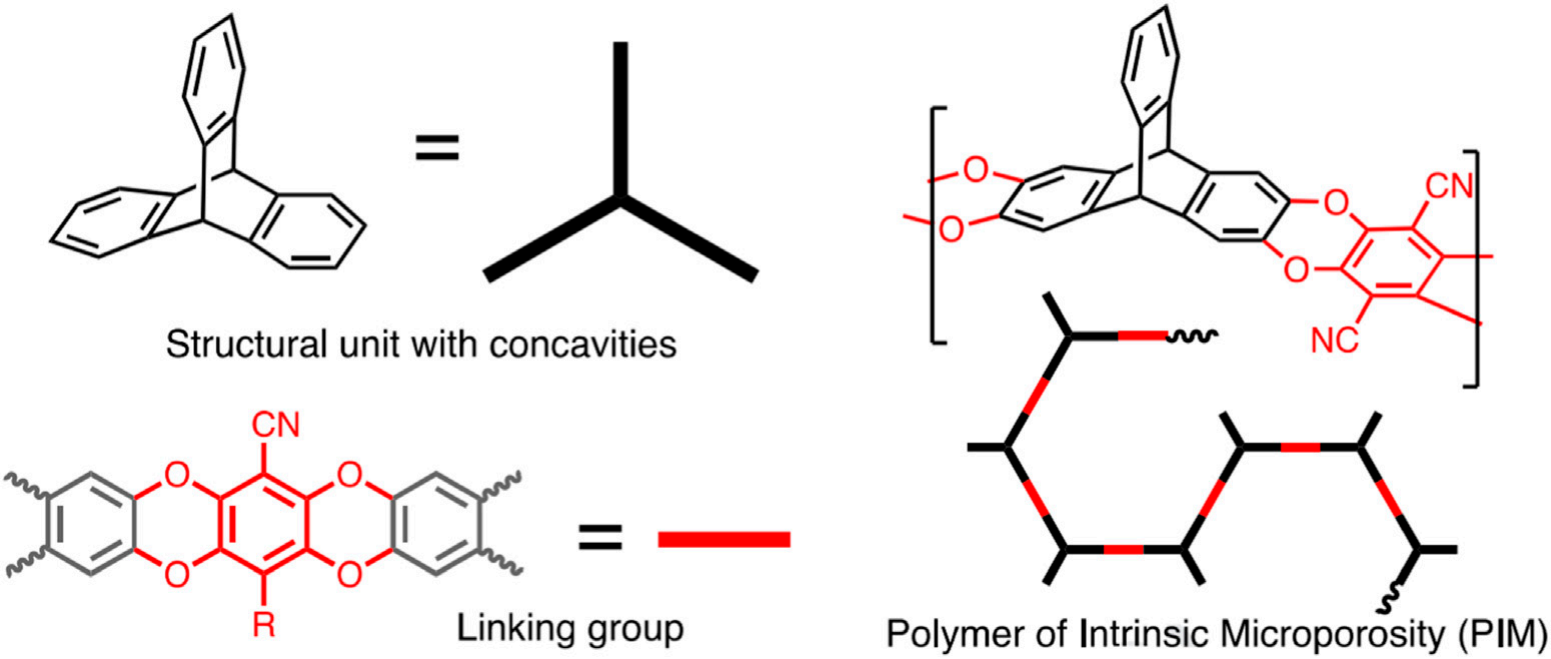
Illustration of PIMs fabrication. Reprinted from (McKeown, 2020) with permission from Elsevier.

PIMs are known as the polymers with amorphous microporosity and rigid main chains. Appropriate solubility in organic solvents, great surface area and their film formation characteristics eventuate in the development of PIMs in industry (McKeown, 2012; Agarwal et al., 2020; Perry et al., 2020; Polak-Kraśna et al., 2021). PIMs are an excellent instance of how changing a co-monomer makes a considerable impact on the porosity of polymer. PIMs are characteristically different from other classes of porous polymeric materials. PIMs have microporosity but do not include designated frameworks (Perry et al., 2020). Figure 2 presents a schematic illustration of the phthalocyanine-based PIM. In an investigation, Budd et al. studied the synthesis process of a hexaazatrinapthylene (Hatn)-based PIM. Based on their investigation (Hatn)-based PIM has a great capability to adsorb approximately 3.9 mmol g^-1^ of the metal complex when exposing to an excess of palladium (II) dichloride in chloroform solution (Budd et al., 2003). Ling et al. fabricated a series of metalized PIMs from a carboxyl-based functionalized PIM (C-PIM). They corroborated that C-PIM-Na demonstrated the greatest CO_2_ capture capacity of 2.44 mmol g^-1^ compared to other metalized PIMs (Ling et al., 2020). Stanovsky et al. conducted an experiment with the aim of purifying flue gas applying the ultra-permeable tetramethyl tetrahydronaphthalene - based PIM coupled with bicyclic triptycene. Acceptable CO_2_/N_2_ selectivity (in the range of 11–18) encouraged the potential application of this PIM for industrial MCS (Stanovsky et al., 2020). Figure 3 schematically depict the process of PIMs fabrication.

The synthesis of PIMs is an important milestone toward the use of this type of POMs in membrane-based CO_2_ separation. PIMs can be synthesized via a polymerization reaction on the basis of a double-aromatic nucleophilic substitution mechanism (Scherf, 1999). This reaction possesses great potential to form two simultaneous covalent bonds with appropriate performance to provide a linking group composed of fused rings (McKeown, 2012). Overally, aromatic nucleophilic substitutions can be of great interest to be done particularly if the halide-including monomer is activated via an electron-withdrawing substituent (i.e., –CN, F, etc.) (Eastmond et al., 2001).

### 2.3 COFs

**FIGURE 4:**
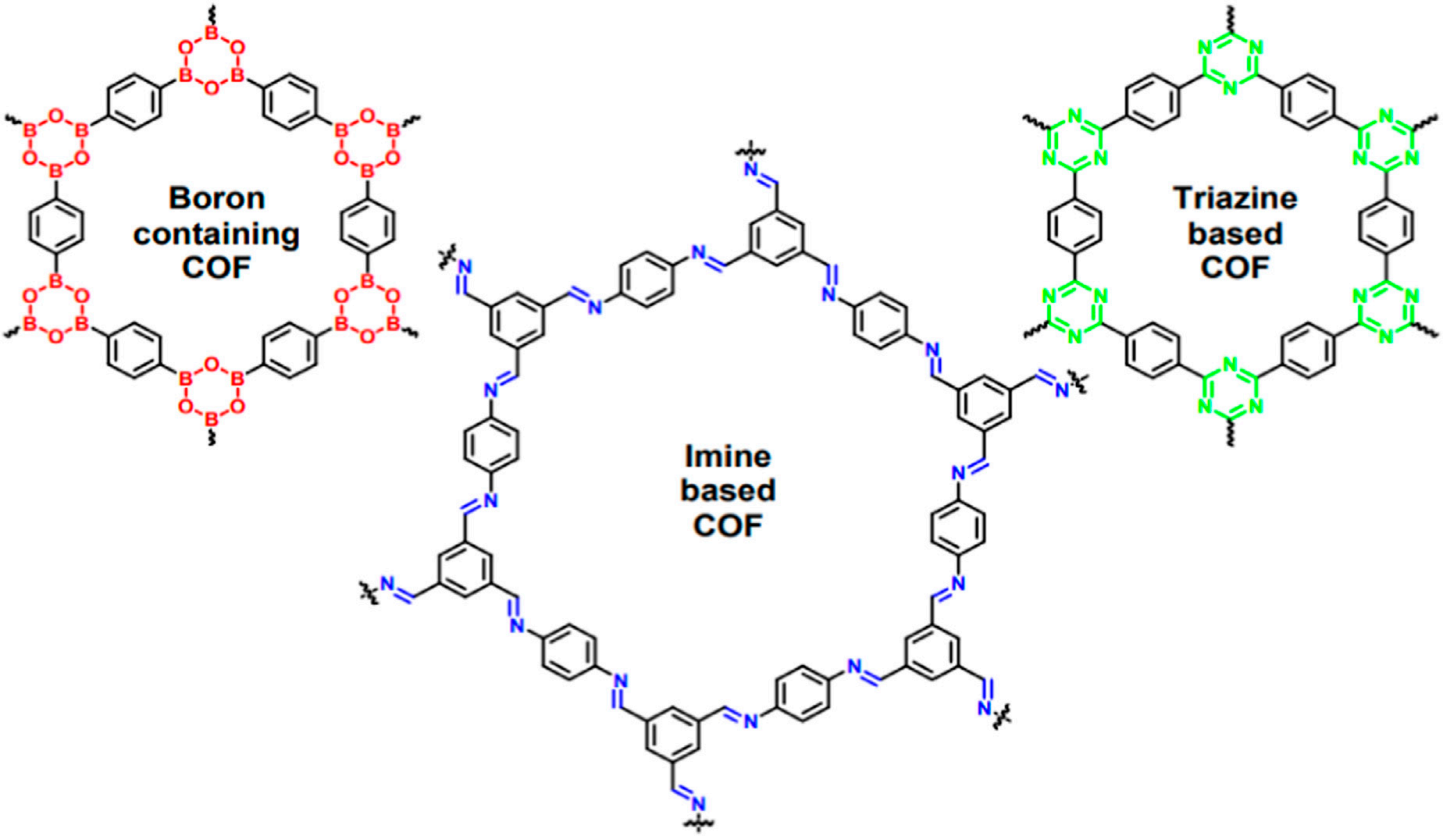
Molecular structure of different COFs (Bagheri and Aramesh, 2021).

COFs are known as one of the significant members of POMs, which are fabricated via molecular-structured blocks interconnected by covalent bonds. Figure 3 depicts the molecular structure of different COFs. Various features such as custom-made properties (achieved by functionalization), structural versatility and uniform pore size distribution have authorized the COFs to be used in an extensive range of applications like membrane-based gas separation and cancer treatments (Guan et al., 2020; Sharma et al., 2020; Guo et al., 2022). The remarkable development of COFs is prominently justified because of their self-healing capability and thermodynamically manageable covalent chemistry, which eventuate in long-range crystalline structure (Pachfule et al., 2018/01). Compared to MOFs, COFs usually have lower density and therefore show outstanding stability in organic solvents. Moreover, COFs have the ability to tolerate harsh situations and maintain their crystallinity. In comparison with inorganic zeolites and porous silica materials, COFs possess greater efficiency due to higher porosity and tunable pore size, which accelerate the penetrant diffusion (Cooper, 2013). In Figure 4, the molecular structure of different COFs is presented.

Noteworthy advantages of COFs like inherent porosity, adjusted channel structure, low density, great stability and designable functionality have made them suitable for application in different scientific fields like CO_2_ separation, optoelectronics, drug delivery and adsorption (Wu and Yang, 2017; Altundal et al., 2020; Ghosh and Banerjee, 2023). In the case of membrane-based CO_2_ separation, porous COFs have been of paramount attentions owing to their brilliant ability for the storage and separation of major greenhouse gases (i.e., CO_2_), H_2_ and methane (Xia and Liu, 2016).

### 2.4 CMPs and CPPs

**FIGURE 5:**
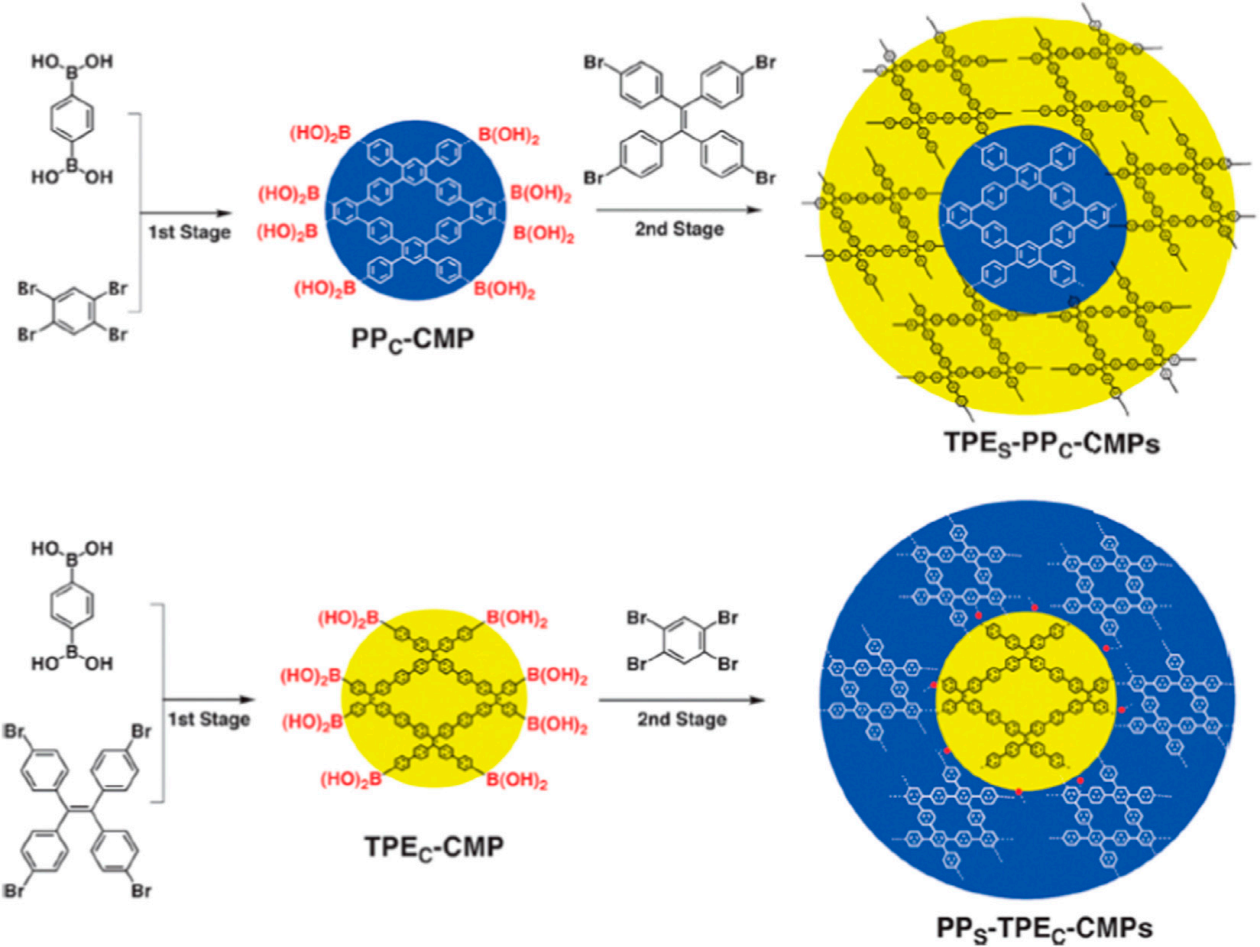
Synthesis process of core−shell structured CMPs. Reprinted from (Xu Y. et al., 2013) with permission from ACS Publications.

CMPs/CPPs refer to an important category of polymeric materials that mix extended π-conjugation with a microporous structure. Generally, CMPs/CPPs are known as microporous polymeric materials but can be accompanied by great amount of mesoporosity (Lee and Cooper, 2020). CMPs/CPPs possess great potential to be extensively used in disparate applications (i.e., separation processes, heterogeneous catalysis, energy storage and so on) owing to their brilliant properties like high porosity, tunable chemistry, appropriate chemical resistance, and thermal stability (Talapaneni et al., 2016; Wang et al., 2017; Liao et al., 2018; Cao et al., 2022). Although gas separation and storage is regarded as the most prevalent area of investigation for CMPs/CPPs, some drawbacks such as the application of expensive transition metals in their synthesis deteriorates their popularity in large-scale separation applications (Lee and Cooper, 2020). Figure 5 represents a schematic demonstration of the synthesis process of core−shell structured CMPs/CPPs. CMPs can be well identified as the most efficient POMs for the separation of CO_2_ greenhouse gas due to their noteworthy advantages including high surface area and tunable properties (Reddy et al., 2021; Zhang et al., 2023). The importance of CMPs is because of the necessary bond conjugation and amorphous morphology. Poly (arylene ethynylene)s were introduced for the first time in 2007 as the synthesized CMPs (Jiang et al., 2007).

### 2.5 CTFs

CTFs can be defined as an important type of organic polymers fabricated by aromatic 1,3,5-triazine rings with planar π-conjugation properties (Wei et al., 2019; Liao et al., 2023). The occurrence of conjugation between aromatic rings and triazine rings significantly declines overall energy of π-conjugated molecules in the frameworks and therefore, significantly enhances the chemical stability. In the majority of cases, N-containing CTFs frameworks have shown their great potential in adsorption/separation and catalysis (Jadhav et al., 2019; Jiang et al., 2019). Most recently, CTFs have been appeared as a promising type of POMs, which is regarded as an adaptable platform for various applications due to their impressive characteristics such as permanent microporosity and appropriate thermal/mechanical stability and chemical resistance even in the strong acidic/basic environment.

**FIGURE 6:**
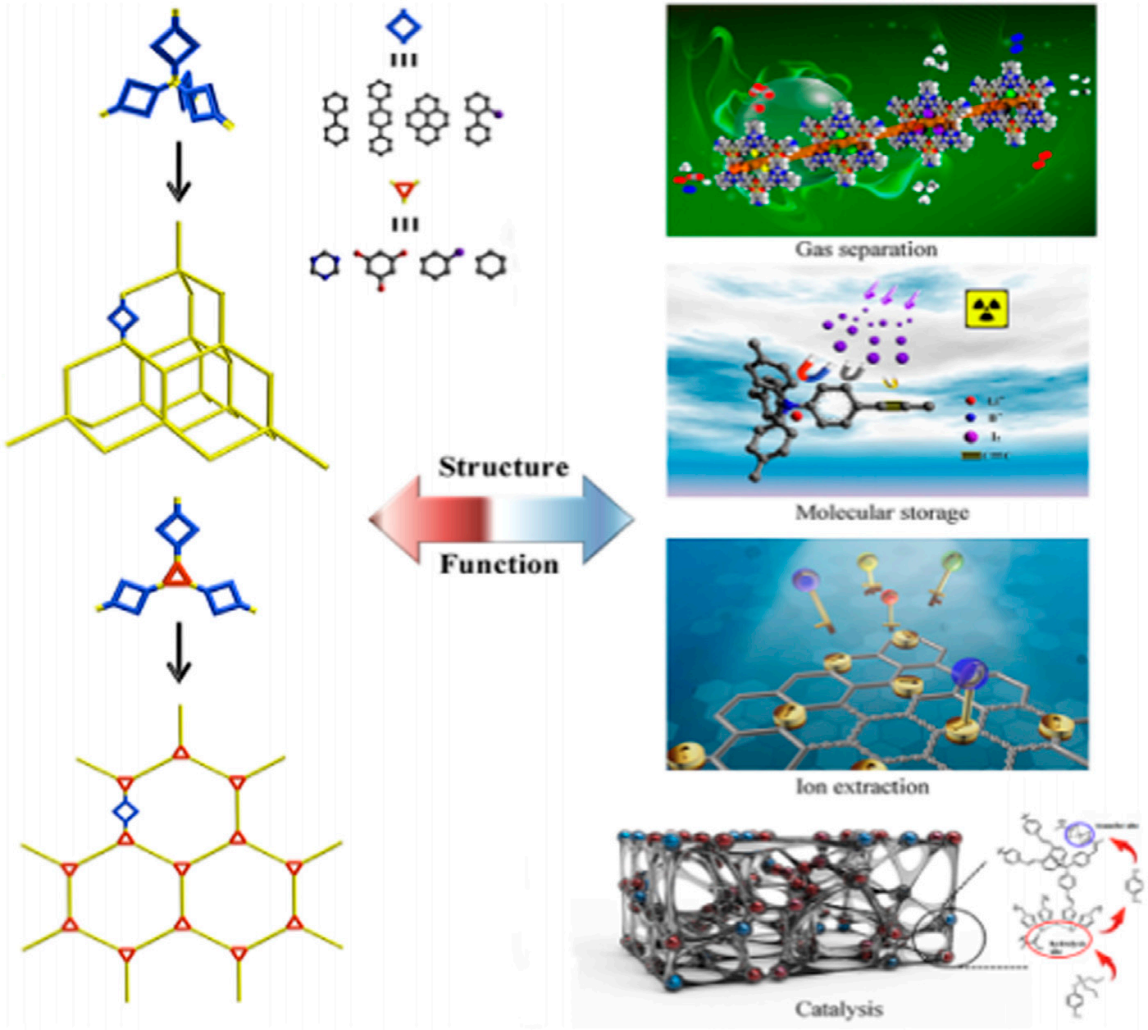
Schematic depiction of PAFs for various industrial applications (Yuan and Zhu, 2019).

The powerful covalent linkages, intrinsically great content of nitrogen atoms and excellent capability to add hetero-atoms in the structural skeleton have made CTFs versatile for numerous potential applications like gas separation and dye adsorption (Krishnaraj et al., 2020). Additionally, they are reproducible and recyclable, which permits them to be a noteworthy candidate in terms of sustainable materials. Figure 6 represents a schematic demonstration of photocatalytic CO_2_ separation from formic acid using CTFs.

### 2.6 Composite membranes

In the recent years, the industrial applications of membrane processes for the effective separation of CO_2_ pollutant owing to their great potential to overcome the negative disadvantages of conventional technologies like cryogenic distillation and desorption (Dai et al., 2023; Yang et al., 2022/03; Shirazian et al., 2020; Faraji et al., 2020). One of the most efficient membrane-based approaches for the separation of CO_2_ is the use of composite membranes. Composite membranes possess brilliant capacity to simultaneously combine the positive points of both inorganic and polymeric membranes, which make them a hotspot for scientific research (Pishnamazi et al., 2020/09). Composite membranes can be considered as mixed matrix membranes (MMMs), which are often used for the efficient separation of CO_2_. Due to the presence of disparate challenges toward the use of inorganic or polymeric membranes, composite membranes have been employed to solve the existed limitations of both aforementioned membranes. The industrial use of MMMs is being significantly enhanced due to their ability to combine the compactness of polymeric membranes and great permeability of inorganic membranes (Saqib et al., 2019).

### 2.7 PAFs

Figure 6 presents the schematic depiction of PAFs and their various applications in industry. PAFs are another category of POMs. PAFs, which are manufactured by carbon-carbon-bond-linked aromatic-based building units (CCBLABU), possess remarkable features such as rigid structures and great surface area. The presence of strong carbon−carbon linkage eventuates in enhancing the chemical resistance of this class of POMs in undesirable chemical environments (Tian and Zhu, 2020). Hence, PAFs illustrate excellent characteristics in chemistry and functionalities in comparison with traditional POMs like zeolites and MOFs. PAFs can be freely functionalized by severe chemical treatments (Tian and Zhu, 2020). Table 2 gives a comprehensive summarization about the characteristics of various POMs investigated in this review paper.

**TABLE 2:** Detailed summarization about the characteristics of various POMs (Bildirir et al., 2017; McKeown, 2017; Huang and Turner, 2018; Liu et al., 2019; Guan et al., 2020; Lee and Cooper, 2020; Sharma et al., 2020; Tian and Zhu, 2020).

POMs	Porosity	Designability	Crystallinity	Stability	Synthetic strategy
HCPs	Micro	Acceptable	Amorphous	Excellent	• PCL
					• DOP
					• ECL
PIMs	Micro	Acceptable	Amorphous	Good	Polymerization reaction based on a double-aromatic nucleophilic substitution mechanism to form the dibenzodioxin linkage
COFs	Micro/Meso	Good	Modest to high	Good	Self-assembly based on covalent bond
CMPs/CPPs	Micro	Good	Amorphous	Excellent	Cross-coupling of building blocks with different geometries
CTFs	Micro	Acceptable	High	Excellent	Trifluoromethanesulfonic acid catalyzed method at room temperature and microwave‐assisted conditions
PAFs	Micro	Good	Amorphous	Good	carbon-carbon-bond-linked aromatic-based building units (CCBLABU)

## 3 POMs for CO_2_ separation: challenges and opportunities

Development of economical/novel technologies for CO_2_ separation from emission sources is considered as the most appropriate strategy to decrease the anthropogenic emissions of this acidic pollutant (Vesovic et al., 1990). POMs have been provided excellent capabilities for CO_2_ separation processes due to their brilliant privileges compared to porous inorganic materials (i.e., zeolite) or inorganic-organic hybrids (i.e., MOFs), such as appropriate stability and chemical robustness to acid and base (Zhang and Dai, 2017). In the recent 20 years, remarkable progressions have been made by the appearance of various POMs such as PIMs, CTFs, COFs, CMPs, HCPs and PAFs (Zhang et al., 2015). Generally, POM synthesis needs particular rigid monomers, which possesses acceptable resistance against the intermolecular packing, and consequently results in high porosity (Hao et al., 2015). POMs with different functionalities have been developed for better physico-chemical and CO_2_ separation properties. A summary of the more significant results is presented in Table 3.

**TABLE 3:** A comprehensive summary of the more significant results in CO_2_ separation.

POMs	Significant results	Ref.
HCPs	Carbazole-based HCPs (CHCPs) possessed greater CO_2_/N_2_ and CO_2_/H_2_ selectivities compared to benzene-based HCPs (BHCPs) and polystyrene-based HCPs (PHCPs) at 1 bar and 298 K. The order is CHCPs > BHCPs > BHCPs	Ramezanipour Penchah et al. (2020)
PIMs	Significant improvement of CO_2_ selectivity from 20.4 to 58.1 by presenting superacid-induced self-cross-linked PIM compared to pristine PIM	Zhou et al. (2020)
COFs	Improvement of CO_2_ permeability by about 2.2 times by applying hollow structured-COF fillers compared to pure Pebax membrane	Liu et al. (2021)
CMPs/CPPs	Great potential of functionalized biphenylene-based CMP for CO_2_ separation from CO_2_/CH_4_ and CO_2_/N_2_ mixtures with maximum adsorption capacity of 87.4 cm^3^ g^-1^ and reasonable CO_2_/N_2_ and CO_2_/CH_4_ selectivities (27.9 and 5.6) at 273 K and 1 bar	Wang et al. (2018)
CTFs	• CO_2_ separation efficiency of 2,4,6-tris(4-cyanophenylamino)-1,3,5-triazine (TAT), 2,4,6-tris(4-cyanophenoxy)-1,3,5-triazine (TOT), and 2,4,6-tris(4-cyanobenzenesulfenyl)-1,3,5-triazine (TST) are proved to depend on the incorporated heteroatoms	Liao et al. (2020)
	• CO_2_ separation efficiency of TAT, TOT and TST improves in the order of PhNH- > PhO- > PhS-linkage in CTFs	
PAFs	• High CO_2_ uptake/selectivity make the nitrogen‐rich PAF promising for CO_2_ separation process	Ben and Qiu (2020)
	• Tailoring the pore diameter of PAFs improves their ability in CO_2_ separation process	

Table 4 gives detailed information about the challenges towards the development of functionalized POMs-based membrane for CO_2_ separation.

**TABLE 4:** Challenges towards the development of POMs-based membrane for CO_2_ separation (Krishnaraj et al., 2020; Prasetya et al., 2019; Johnson et al., 2008; Haq et al., 2021; Fouladivanda et al., 2021; Yang et al., 2020/03).

POMs-based membrane	Challenges towards material selection	Challenges towards membrane fabrication/module configuration	Challenges towards membrane performance
Mixed matrix membranes (i.e., POFs, PIMs)	• Selection of resistant fillers (i.e., ionic liquid (IL)-modified UiO-66-NH_2_) against water vapor, pollutants and so on	• Optimization of particle loading	• Addition of nanoparticles such as Ni-ZIF-8 to enhance penetrant membrane interactions
	• Selection of appropriate polymers to tolerate unfavorable operational conditions	• Development of novel synthesis/characterization method to enhance the efficiency	• Development of environmentally-friendly POMs-based membranes with low environmental toxicity
		• Fabrication of asymmetric membrane in a hollow fiber formation	• Optimization of operational conditions
Pure POFs membrane	• Selection of resistant POFs against unfavorable operational conditions	• Advancement of hollow fiber formation	• Decreasing the thickness of membrane to enhance permeation
		• Attention of polymeric substrate as a more economical option for membrane support	• Aging testing performance
Microporous polymers (i.e., CTFs, HCPs, PAFs)	• Selection of resistant polymers against unfavorable operational conditions	• Advancement of hollow fiber formation	
		• Fabrication of nitrogen, oxygen, and fluorine-doped carbon molecular sieve membranes	
		• Attention of low-temperature thermal adjustment for membranes	

## 4 Conclusion and future directions

This article presents a review about the possibility of POMs application in CO_2_ separation processes. Critical investigation on the functionalization features of different classes of POMs including HCPs, PIMs, COFs, CMPs, CTFs, and PAFs is implemented and a detailed summarization about the characteristics of various POMs such as porosity, designability, crystallinity, stability, and synthetic strategy is presented to clarify the advantages and disadvantages of these materials in CO_2_ separation. Specially, reviewing the state-of-the-art applications of functionalized POMs in CO_2_ separation and the challenges and future directions towards the correct perception of POMs are presented to find the existing research gaps in this field. One of the prominent challenges towards the application of POMs is the perception of their promising efficiency in industrial-based CO_2_ separation processes. Several challenges are existed that their interpretation seems to be mandatory. For instance, fabrication of POMs-based membrane in hollow-fiber configuration may be a good choice to improve the efficacy and components interaction. Appropriate optimization of POMs-based membranes’ operational condition is regarded as another matter. Optimum operational conditions (i.e., pressure and temperature) must be precisely evaluated, especially for studying CO_2_-induced plasticization. Environmental consideration and fabrication of environmentally-friendly POMs-based membranes’ with the minimum detriments and toxicities is an important challenge towards the development of these types of polymeric membranes, which must be thoroughly investigated. Eventually, investigation of membrane aging must be in the priority of evaluation due to its significant impact on the long-term performance of POMs. More comprehensive investigation in the development of emerging POMs for MCS process is definitely required. The study not only should not be conducted in discovering new POMs but also must be towards optimizing the currently developed polymers due to their promising efficiency in CO_2_ separation. Furthermore, an extensive economic feasibility analysis is needed to be implemented to evaluate the possibility of POMs application in different industries. If the economic feasibility analysis justifies the use of POMs-based membranes in industries, this technology possesses the potential to replace the traditional methods contributing in efficacious CO_2_ separation processes.
